# Mature Teratoma of the Temporal Bone in 3.5-Month-Old Baby Girl

**DOI:** 10.1155/2015/372089

**Published:** 2015-04-06

**Authors:** Alshema Alqurashi, Essa Bakry, Marta Straube, Christian H. Rickert, Parwis Mir-Salim

**Affiliations:** ^1^Department of Otorhinolaryngology and Head and Neck Surgery, Vivantes Hospital Friedrichshain, Berlin, Germany; ^2^Department of Pathology, Vivantes Hospital Friedrichshain, Landsberger Allee 49, 10249 Berlin, Germany

## Abstract

Mature teratoma is a benign germ cell tumor rarely located in the temporal bone. We are reporting a case of a mature teratoma of the temporal bone in a healthy borne 3.5-month-old baby girl with a 2-day suggestive history of otitis media and polypoidal mass expulsing from the external auditory canal of the left ear. A definitive diagnosis is made after complete excision and histological examination of the tissue. Total surgical excision of the tumor is the treatment of choice.

## 1. Introduction

Teratomas are germ cell tumors that presented in childhood. Although there are rarely presented in the temporal bone, teratoma should be considered in differential diagnosis of any mass lesion in the temporal bone. Early diagnosis and treatment through complete excision of the tumor are recommended to prevent transformation into immature cell and prevent recurrence. Imaging is obligatory not only for evaluation of the temporal bone structures but also for excluding intracranial erosions. According to our knowledge there are only few cases reported about temporal bone teratomas that were successfully treated surgically with no evidence of recurrence.

## 2. Case Presentation

A 3.5-month-old baby girl with normal prenatal, natal, and postnatal history was presented to our emergency room with a 2-day history of otorrhea and 1-day history of a protruded mass from the external auditory canal. There was no fever or changes in feeding habits.

**Figure 1:**
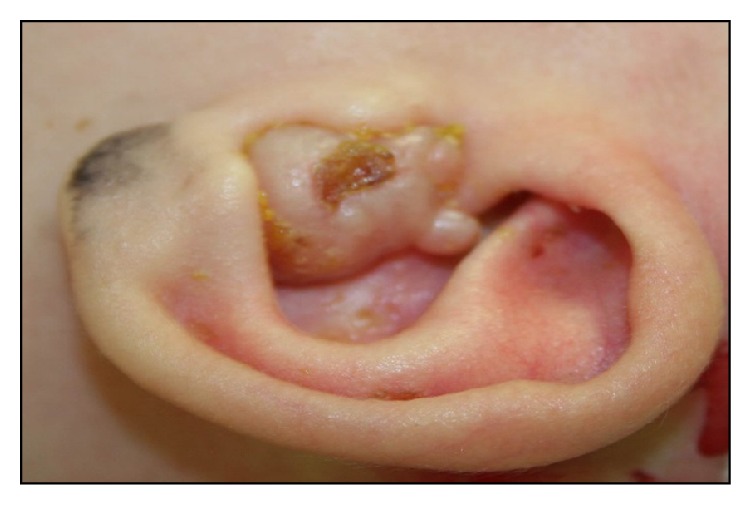
The external auditory meatus of the left ear shows that the external auditory meatus of the left ear was filled out by a nonhemorrhagic polypoidal mass.

Examination revealed a vitally stabile, well-fed baby girl. The external auditory meatus of the left ear was filled out by a nonhemorrhagic polypoidal mass, which was firm in consistency and nontender on palpation (Figure 1). There was also minimal discharge. The tympanic membrane was not visible. Systemic evaluation including cardiovascular and neurological examinations showed no abnormalities.

**Figure 2:**
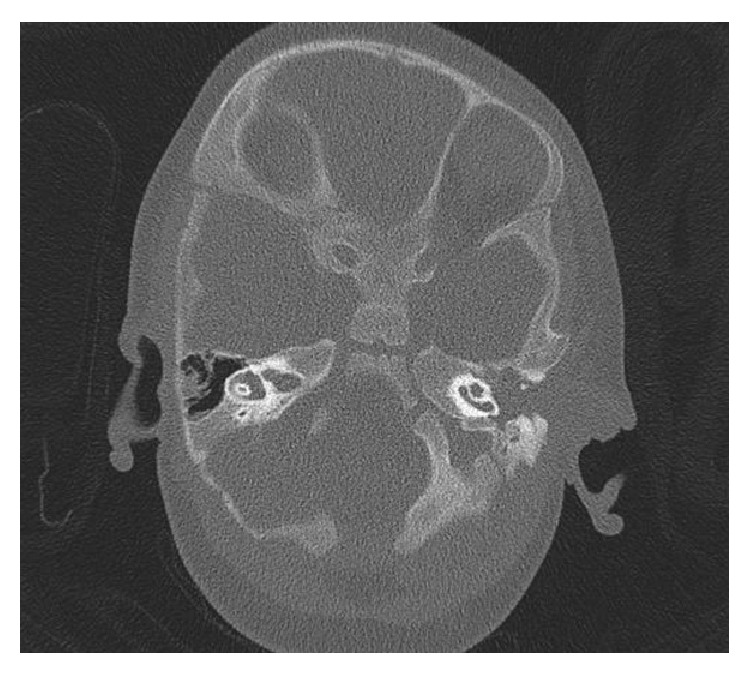
Head CT scan (axial) shows large homogeneous in the left temporal bone.

**Figure 3:**
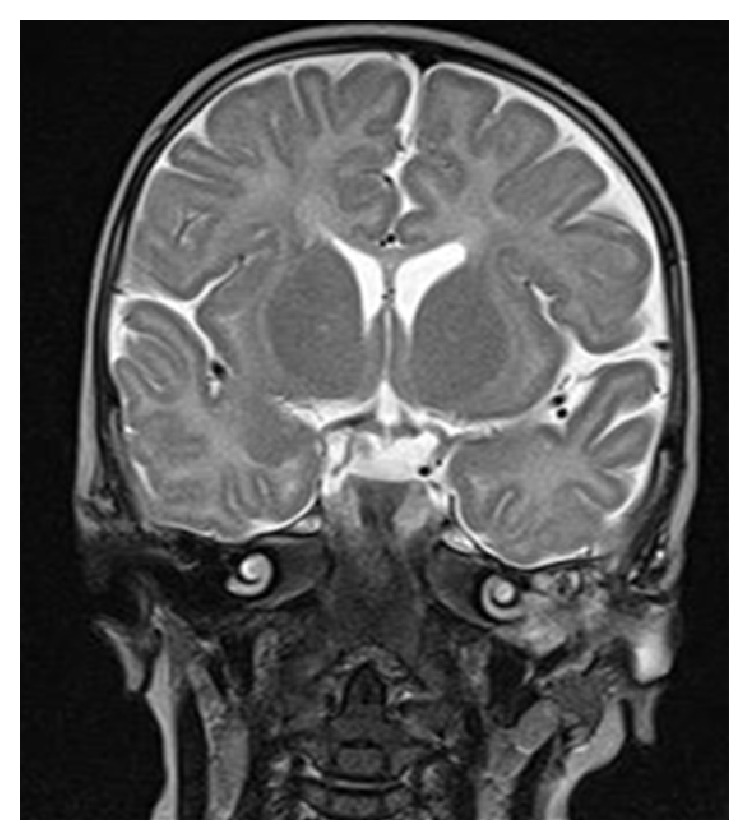
Head MRI T2 (coronal).

**Figure 4:**
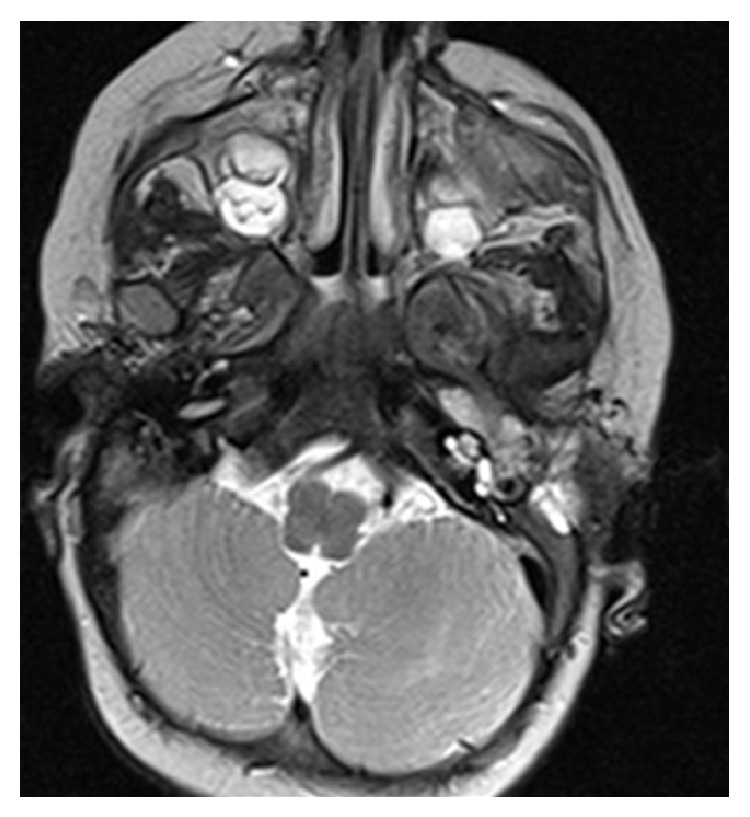
Head MRI T2 (axial).

Radiological evaluation was made which includes CT scan (Figure 2) which revealed a large homogeneous mass (about 9 × 9 × 3.5 cm) in the left temporal bone with protrusion into cavum tympani and the mastoid. The skull base was intact. Magnetic resonance imagining (Figures 3 and 4) with gadolinium revealed a mass of intermediate signal in T2- and hypointense in T1-weighted imaging. This mass showed minimal homogeneous enhancement. There was no sign of diffusion impairment. Inner ear, the brainstem, and cerebellum structures have been without any significant pathological changes.

The fourth ventricle was formally configured, being basal cisterns-free with normal shape and configuration of basal ganglia and cortical and subcortical structures unremarkable.

There is no evidence of calcification or fatty component seen in CT and MRI.

There is no diffusion restriction and ADC is in the normal limit.

Decision was made to take the patient to operating room for exploration and excision. Intraoperatively retroauricular incision was made. The tumor was encountered after drilling the outer cortex of the mastoid and removed by tympanomastoidectomy. The ossicles were dislocated. Facial canal, internal carotid artery canal, and the bulb of the internal jugular canal were intact. There was no skull base defect. The remaining ossicles were removed and tympanoplasty type 3 was performed. Then the incision was closed in 2 layers.

The histopathological study showed a keratinized squamous epithelium tissue with skin appendages, in the form of hair follicles and sebaceous glands, connective tissue, fat cells, and sweat glands. In addition, there are an elastic cartilage and smooth muscle portion. So based on the result, the diagnosis of temporal bone teratoma was concluded.

## 3. Discussion

Teratomas are germ cell tumors that are composed of multiple cell types derived from one or more of the 3 germ layers (ectoderm, endoderm, and mesoderm) [1]. In 1863, Virchow had published his first book about tumors; according to Hamilton, Virchow was the first one who used the Greek word “teraton.”

Abnormal differentiation of fetal germ cells that arise from the fetal yolk sac is believed to be the origin of teratomas. Normal migration of these germ cells may cause gonadal tumors, whereas abnormal migration produces extragonadal tumors [2].

They are most commonly found in sacrococcygeal region (57%) and gonadal regions (29%). Mediastinal teratomas represent only 7% of the tumor whereas head and neck teratoma represents 5% [1, 2]. The sites of involvement of head and neck teratoma include the neck, oropharynx, nasopharynx, orbit, and paranasal sinuses, and rarely temporal bone [3].

Histologically, they are classified into mature and immature teratomas. This classification depends on the presence of immature neuroectodermal elements within the tumor [2]. As in our case, there were no immature cells found in previously reported cases.

Clinically, the patient presents at birth or in early childhood with no sexual predominance [4]. They are rapidly growing tumors and their symptoms are varying depending on their extension. They range from otitis media, and deafness, to facial paralysis or severely neurological deficit due to invasion in the brain tissues.

As shown in the literature the most common presenting symptoms are hearing loss [5–8] and otorrhea [7, 8]; facial paralysis and neurological deficit are rarely presented.

Khan et al. have reported a case in 2013 of a 11-year-old girl with a left side facial palsy due to invasion of the teratoma from the temporal bone into cerebellopontine angle, which was successfully removed through a transtemporal approach [5].

Radiological studies including temporal bone CT scan and MRI are well-established technique to evaluate the temporal bone structures. CT scan is the method of choice in evaluating the temporal bone pathology, skull base erosion, whereas MRI is important in internal auditory canal, inner ear, cerebellopontine angle, and brainstem lesions [9, 10]. Radiologically teratoma appears in the temporal bone usually as an inhomogeneous lesion with the presence of fat, bone, cartilage, soft tissues, and enhancement of the capsule with or without bone destruction [3].

The differential diagnosis of a temporal bone mass should include cholesterol granulomas, petrous apex cephalocele, meningioma, cholesteatoma, rhabdomyosarcoma, chondroma, mucocele, and metastatic neuroblastoma. Cholesterol granulomas are cysts filled with viscous fluid, granulation tissue crystals, and cholesterol and enclosed with a thick fibrous capsule. In MRI, they are hyperintense on T1 and T2 and are not enhanced with contrast material [11].

As shown in previous case studies the only curative treatment is a complete surgical excision of the tumor [5–8]. Early removal of it prevents immature transformation and further extension. The microsurgical approach depends on the extension and the location of the tumor. According to our knowledge there was no need for adjuvant therapy and there was no recurrence in all published cases.

Cholesteatoma appears in CT as a nonenhancing, expansile lesion with variable degree of bone destruction; otherwise in MRI, they have an intermediate to low signal intensity on T1, and in T2 they have a high signal intensity with no enhancement with a contract material.

In CT rhabdomyosarcomas show an aggressive soft tissue mass that causes osseous destruction. They have an intermediate signal intensity on T1, variable signal intensity on T2, and variable enhancement [11]. Chondromas have an aggressive behavior in pediatrics. They appear in CT as a destructive soft tissue mass with tumor calcification in the chondroid variant of the tumor. In MRI they tend to be hypointense on T1 and hyperintense on T2.

As shown in previous case studies the only curative treatment is a complete surgical excision of the tumor [5–8]. Early removal of it prevents immature transformation and further extension. The microsurgical approach depends on the extension and the location of the tumor. According to our knowledge there was no need for adjuvant therapy and therewas no recurrence in all published cases.

## 4. Conclusion 

In our case, clinical examination and radiological imaging have led to the diagnosis of a mature teratoma of the temporal bone. This was confirmed after histological examination of the specimen after the complete successful surgical removal by canal wall down technique.
